# Mr. Ceely on the Nitrate of Silver

**Published:** 1829-10-01

**Authors:** 


XXXI.

NITRATE OF SILVER.

The following observations on this powerful medicine were contained in a letter from Mr. Ceely, an intelligent surgeon in the country.

"In two cases of very painful digestion I used the nitrate for two months, and arrived at four-grain doses. One of the patients lived on gruel two months, and subsequently small portions of fat bacon for three more; and her perseverance has gained for her that health and comfort which she had not enjoyed for five years before. In numerous other cases I have derived infinite advantage from this sedative, conjoined with the plan of diet you have so ably advocated. I have never uninterruptedly continued the use of the nitrate beyond two months; nor have I ever seen any of its ill effects on the skin. I have found this article of great use, made into a pill with extract of poppy or hemlock, or any thing similar, and introduced up the rectum in the quantity of from one to three grains, twice or thrice a day, in that simple tenesmus supervening on active or protracted diarrhœa. Its effects are more decisive and more permanent than the direct sedatives usually employed.

"I have not long since had a successful case of delirium tremens in a female, in which 485 grains of extract of opium was taken in six days; and still more recently, in a more complicated and intermittent case of the disease, 13 grains of the acetate of morphium were taken in six hours, before sleep was procured." R. C.

